# The Genetic Regulation of Infant Immune Responses to Vaccination

**DOI:** 10.3389/fimmu.2015.00018

**Published:** 2015-02-02

**Authors:** Melanie J. Newport

**Affiliations:** ^1^Division of Clinical Medicine, Brighton and Sussex Medical School, Brighton, UK

**Keywords:** SNPs, transcriptional profiling, candidate gene, GWAS

## Abstract

A number of factors are recognized to influence immune responses to vaccinations including age, gender, the dose, and quality of the antigen used, the number of doses given, the route of administration, and the nutritional status of the recipient. Additionally, several immunogenetic studies have identified associations between polymorphisms in genes encoding immune response proteins, both innate and adaptive, and variation in responses to vaccines. Variants in the genes encoding Toll-like receptors, HLA molecules, cytokines, and cytokine receptors have associated with heterogeneity of responses to a wide range of vaccines including measles, hepatitis B, influenza A, BCG, *Haemophilus influenzae* type b, and certain *Neisseria meningitidis* serotypes, amongst others. However, the vast majority of these studies have been conducted in older children and adults and there are very few data available from studies conducted in infants. This paper reviews the evidence to date that host genes influencing vaccines responses in these older population and identifies a large gap in our understanding of the genetic regulation of responses in early life. Given the high mortality from infection in early life and the challenges of developing vaccines that generate effective immune responses in the context of the developing immune system further research on infant populations is required.

## Introduction

Although infant and under-5 mortality rates are reducing, nonetheless 6.6 million children died in 2012 (1). Infectious diseases were responsible for approximately half of these deaths and the majority occurred in low or middle income countries (LMICs). There are no reliable data on morbidity for children at the global level so the burden of disability such as deafness and epilepsy following meningitis, for example, is unknown but likely to be high. Whilst vaccines are now available for many infections and increasingly administered in developing countries, there remains an urgent need to develop or improve vaccines for this age group. The reasons for this are multiple: there are no vaccines yet for some important infections such as malaria and respiratory syncytial virus; vaccines for other diseases need improving or replacing (e.g., BCG); global differences in microbial epidemiology may render effective vaccines developed in one region ineffective in another [e.g., the human papillomavirus (2)]; finally vaccines that do not cover all strains of a pathogen may drive changes in the microbiological epidemiology (or resistance to the strains that are included) that may render the vaccine ineffective. This has been widely discussed in the context of the pneumococcal vaccines (3).

However, despite these challenges, alongside the biological challenges of developing vaccines for infants with immature immune systems that contribute to their increased susceptibility to infection in the first place, there are strong reasons to investigate immune responses in the age group. In order to inform the development of novel or improved vaccines better understanding of the essential pathways involved in immunity to various pathogens is key. Genetics has over the years proved to be a useful tool with which to dissect out immune responses. Genetic studies of the monogenic primary immunodeficiencies revealed critical roles for a number of genes in protective immunity (e.g., mutations in genes encoding components of the interferon-gamma/interleukin-12/23 pathways predispose to disseminated non-tuberculous mycobacterial infections, highlight the important of this pathway in mycobacterial immunity more broadly (4, 5).

## Role of Host Genetics in the Regulation of Vaccine Immune Responses

Most immune responses, like many biological responses, show wide-ranging inter-individual variation within in a population, whether in response to vaccination or natural infection. For example, interferon-gamma (IFN-g) responses following neonatal BGC in the Gambia follow a normal distribution (6) and 68% of variation in *in vitro* tumor necrosis factor (TNF) responses to microbial components measured in whole blood samples collected from a Ugandan population was genetic (7). Such patterns indicate a multifactorial etiology where both genes and environmental factors interact. Heritability studies can then be used to estimate the magnitude of genetic contribution to this phenotypic variation. It should be remembered however, that heritability estimates are specific to the population that was studied and they do not indicate how many genes may be involved or what the underlying genetic model is, i.e., whether there one major gene whose effects are modified by a few minor genes, or many genes each with a minor impact. Studies have shown that responses to a number of vaccines are heritable. These include BCG (8), hepatitis B virus (8–10), *Haemophilus influenzae* type b (11), tetanus toxoid (8), pneumococcal polysaccharide vaccine (12, 13), varicella vaccine (14), measles, mumps, and rubella (15, 16). Selected data from these studies are presented in more detail in Table 1.

**Table 1:** **A summary of studies that have demonstrated heritable immune responses to vaccines**.

Study	Study site and age group	Vaccine	Immune response studied	Heritability (%) (95% CI)
Newport et al. (8)	The Gambia, infant twins	BCG	IFN-g/PPD	41 (10–71)
			IL-13/PPD	46 (5–75)
			IFN-g/KMTB	39 (3–71)
			IL-13/HSP65	50 (29–67)
		Hepatitis B	Anti-HBs Ab conc.	77 (63–85)
		Tetanus	Anti-TT Ab conc.	44 (16–70)
			IL-13/TT	64(50–75)
		Polio	Neutralizing Ab conc.	60 (43–73)
		Diphtheria	Anti-DT Ab conc.	49 (17–77)
		Pertussis	IFN-g/PER	53 (35–67)
			IFN-g/FHA	65 (50–76)
			IL-13/PT	57 (40–71)
		N/A	Total IgG conc.	78 (67–85)
Hohler et al. (9)	Germany, adult twins	Hepatitis B	Anti-HBs Ab conc	61 (41–81)
Yan et al. (10)	China, 1-year-old twins	Hepatitis B	Anti-HBs Ab conc	91 (76–97)
Lee et al. (11)	Gambia, infant twins	Hib	Anti-PRP Ab conc.	51 (95 CI: 32–66),
Klein et al. (14)	United States, 12- to 23-month-old siblings	Varicella	Anti-VZV Ab conc.	45 (15–75)
Tan et al. (17)	United States, 2–18-year-old twins	Measles	Anti-measles Ab conc	88.5 (52.4)
		Mumps	Anti-mumps Ab conc	38.8 (1.6)
		Rubella	Anti-rubella Ab conc	45.7 (4.9)
Konradsen et al. (12); Konradsen et al. (13)	Denmark, Adult twins	Pneumococcal polysaccharide (Pneumovax R)	8 specific IgG anti-pneumococcal Ab concs	F-test values between 2.94 and 7.11

*CI, confidence intervals; BCG, Bacille Calmette-Guerin; IFN-g, interferon-gamma; PPD, purified protein derivatives; IL-13, interleukin-13; KMTB, killed *Mycobacterium tuberculosis*; HSP65, heat shock protein 65; HBs, hepatitis B surface antigen; Ab, antibody; conc., concentration; TT, tetanus toxin; DT, diphtheria toxin; PER, pertactin; FHA, filamentous hemagglutinin; PT, pertussis toxin; IgG, immunoglobulin G; Hib, *Haemophilus influenzae* type b; PRP, polyribosyl ribitol phosphate; VZV, varicella Zoster virus*.

*(1) Only lower confidence intervals were reported in this study; (2) The *F*-test was used in this study to compare intrapair correlations between monozygous and dizygous twins. An *F*-test score of >2.86 is considered significant – in this case the antibody responses following pneumococcal vaccination were significantly more highly correlated in the MZ twin pairs when compared to the DZ twin pairs*.

## Gene Identification

There have been many scientific advances in genetics, genomics, and the accompanying technology to allow high throughput data generation that are being widely applied in the hunt for genes that regulate vaccine responses. Historically, before the human genome sequence was available – which in turn led to genome-wide association studies (GWAS), whole genome sequencing (WGS), whole exome sequencing (WES), and gene expression profiling to mention a few – most investigators used a candidate gene approach to detect genetic variations associated with immune responses. The frequencies of allele variants within a gene hypothesized to be involved in response regulation were correlated with the magnitude of the immune response following vaccination within populations. Many associations have been reported and a selection summarized in Table 2, which is by no means comprehensive but intended to give a range of the studies that have been done, the vaccines studied and the number of putative genes identified. It can be seen that more studies have been published for some vaccines such as measles [reviewed by Haralambieva et al. (18)] and hepatitis B, but it is important to realize that there is publication bias for genetic association studies and negative findings are less frequently reported. With the exception of the BCG study, which used T cell cytokine responses as the phenotype, all the studies included in Table 2 use antibody responses as the phenotype with the goals of trying to understand why vaccines are poorly immunogenic in some but not all individuals and to identify genetic factors associated with persistence often immune response. Of note, all except two the studies included in Table 2 were conducted in older children and adults. The BCG study was conducted in South African neonates (19) and one of the studies showing an association between Class II HLA and failure to respond to hepatitis B vaccination was conducted in neonates in Italy (20). A more comprehensive systematic review of the link between genetic variation and variability in vaccine responses identified over 2500 potentially relevant studies in the initial search (in July 2013) of which 70 were considered in more detail and 34 fully analyzed (21). However, it should be noted that only one of the studies reviewed was conducted in infants.

**Table 2:** **Some reported associations between variants in candidate genes and immune responses to vaccines**.

Vaccine to which immune response was measured	Gene(s) associated with vaccine response	Reference
Measles	*HLA, TLR2–6, DDX58, OAS1, ADAR*	(22–25)
Hepatitis B	*IFNG, MAPK8, IL10RA ITGAL, IL4, IL4R, IL10, HLA, TNF, IL12B*	(20, 26–31)
Conjugated pneumococcal	*IL4, IL4RA, IL13*	(32)
Group C meningococcal	*TLR3, CD44*	(33)
Influenza	*HLA*	(34)
Hib	*TIRAP*,	(35)
Hepatitis A	*IL10*	(36)
Rubella	*HLA, LTA, TNF, LST1*	(37, 38)
Diphtheria	*IL10*	(28)
Tetanus	*IL4RA*	(28)
BCG	*TLR1, TLR6*	(19)

*HLA, human leukocyte antigen; TLR, toll like receptor; DDX58, dead box polypeptide 58; OAS1, 2-prime, 5-prime oligoadenylate synthetase 1; ADAR, RNA-specific adenosine aeaminase; IFNG, interferon gamma; MAPK8, mitogen-activated protein kinase 8; ITGAL, integrin alpha L; IL, interleukin; IL-4R, interleukin-4 receptor; TNF, tumor necrosis factor; IL-12B, interleukin-12 beta chain; CD44, cluster of differentiation 44; IL-4RA, interleukin-4 receptor alpha chain; TIRAP, TIR-containing adaptor protein; LTA, lymphotoxin; LST1, leukocyte specific transcript 1*.

One of the limitations of the candidate gene population association approach in the past has been that often studies were small and therefore underpowered, especially if numerous genetic variants were being tested in the same small cohort. Furthermore, results were rarely reproducible between groups and populations. There are a few exceptions: for example meta-analyses for hepatitis B vaccine responses found evidence that variants in class II HLA and interleukin-4 (IL-4) were significantly associated with antibody responses (31, 39). As technology advanced it became possible to test multiple candidate gene variants in much larger sample sizes and large scale experiments such as that by Davila et al. became possible (29). In this study, 6091 single-nucleotide polymorphisms (SNPs) in 914 immune response genes were typed in 918 Indonesian people in a search for variants associated with hepatitis B vaccination responses. Previous associations with class II HLA were confirmed and a new association was identified with a SNP in the *FOXP1* gene, a transcription factor involved in B-cell development.

As the field further developed, and the human genome sequence was first published (40), then the extent of variation within it was captured (41), it became possible to systematically interrogate genetic variants (mainly SNPs) across the genome and results could then be analyzed against phenotype data – this was the introduction of the GWAS. There was also a need to develop capacity to store and statistically analyze such large datasets. Over 500,000 SNPs could be typed in several thousand individuals (42) to identify novel diseases susceptibility loci.

A number of GWAS studies have been undertaken or are underway for childhood vaccines including MMR (43). In this study, SNPs in two genes were associated with the magnitude of IFN-g responses to rubella vaccination in school age children. Although not part of Expanded Program on Immunization schedules for children, GWAS have been conducted to detect associations with immune responses to smallpox vaccination, where significant associations were identified within a number of cytokine gene variants (44, 45), and anthrax vaccination where suggestive rather than significant associations was found with SNPs located with the HLA class II loci, the mex-3 homolog C (*MEX3C*) gene and the splA/ryanodine receptor domain and suppressor of cytokine signaling (*SOCS*) box containing 1 (*SPSB1*) gene (46). Genome-wide linkage studies identified three loci on three different chromosomes found to be linked to BCG responses in a Gambian twin cohort described elsewhere (8) and a GWAS has been undertaken in this cohort as well.

Looking forward, technology has developed in other parallel disciplines (or other “omics” as they are increasingly referred to) allowing a much more integrated and holistic approach toward the goal of developing new and more effective vaccines that work in everyone. Indeed, the term vaccinomics was coined by Poland and colleagues to capture the range of technologies now available to investigate the complex biological system responsible for providing protective immunity (47).

Genome-wide expression profiling took studies to the next level, investigating the activity of the genome rather than the inherent genome variation, i.e., which genes are “switched on” in any given situation such as stimulation post vaccination. In one study, microarray transcriptional profiling was used to assess responses to yellow fever vaccine (48). Sixty five of the 97 differentially regulated genes that were studied were shown to correlate with antibody and CD8 T cell specific responses and a subset of these could predict the response to vaccination. Lu et al. used a combination of transcriptional and epigenetic profiling to study both gene expression and methylation patterns in 25 infants who had received hepatitis B vaccine and were know to be either high or low responders according to their antibody responses (49). This study showed that modifications through hypo/hypermethylation, down regulation, and post transcriptional control were associated with low response to hepatitis B vaccine. There is also scope to harness *in silico* advances in bioinformatics, computational modeling and pathway analysis to enable the prediction of signature immune responses reviewed in more detail by Poland et al. (50).

Cutting edge technology is also being applied to enable more detailed and sophisticated phenotyping of the immune response to vaccination, which is currently relatively crude, involving the measurement of antibody levels and for some vaccines cellular responses through measuring cytokine levels. A recent study used high throughput sequencing technologies to characterize in detail the B-cell receptor repertoires in adults who had received a conjugated Hib/MenC/tetanus vaccine (51). This approach allowed the identification of antigen specific sequences that could be represent a welcome improvement in the ability to measure vaccine immunogenicity in a meaningful way.

## Conclusion

There is good evidence that host genetic factors are important, but not sole, determinants of responses to vaccination. Initial genetic epidemiology studies demonstrated many responses were heritable, and using the genetic tools available at the time a number of groups went on to show associations between candidate genes and specific responses. Momentum in the field increased exponentially in the last decade or so due to advances in scientific methodology and supporting technology, which has allowed large scale interrogative studies. The studies referred to above by Querec et al., Lu et al., and Truck et al. illustrate the potential new technologies bring toward unraveling the complexities of immune responses to vaccines and it is likely that the field will advance rapidly. This has obvious implications for the development of better vaccines, and equivalent studies in infectious diseases for which there are no vaccines could help identify pathways critical to the immune responses that could be targeted in vaccine development.

## Conflict of Interest Statement

The author declares that the research was conducted in the absence of any commercial or financial relationships that could be construed as a potential conflict of interest.
